# Event and Comment

**Published:** 1908-11-15

**Authors:** 


### DENTAL REGISTER.

**Vol. LXII. November 15, 1908. No. 11.**

### EVENT AND COMMENT.

### New Constitution for the National Dental Association.

The editor of *Items of Interest* prints in his September issue the constitution which he introduced at the recent meeting of the National, and asks for opinions on the desirability of its adoption. It is substantially the constitution and by-laws of the American Medical Association adapted to the needs of our National Organization. The proposition is too great for us to print entire in our pages. We fear its adoption would provide for so many new and different functions that the Association's work would be hindered rather than facilitated for a time. The whole scheme seems to be, to materially increase the membership of the Association, and add to its revenues, for the purpose of securing the necessary funds to print an Association Journal. Our first impression of the document, is that it is so intricate and complicated that it will require a goodly number of the most capable men in our profession to administer it at all, and the probability is that it will cause considerable confusion before this is accomplished. Can't it be made simpler and be just as effective? The intent is to eliminate all political influence that is now supposed to stand seriously in the way of making a great National and representative body. This is to be accomplished by delegating to a certain number of men called the "House of Delegates" all the business matters of the Association. This body is constituted by representatives of different societies and members and shall not exceed one hundred and fifty and may be less as an equitable apportionment may demand. No member of this "House of Delegates" can be elected to any other office. This "House of Delegates" elects the officers of the Association, and transacts all business, either by the nine trustees which it elects, or through its other officers. This is equivalent, practically to making the "House of Delegates" the executive committee with full powers to transact all the Association's business. It is only an executive committee, and as it is compelled to select presiding officers from without its own numbers, there is good reason for believing that this will eliminate unworthy strife for the honorable places. The manner of apportionment of delegates is to be left to a committee. Would it not be much wiser to specify that every state society should be entitled to one delegate, with a provision that for every three hundred members in excess of two hundred it shall be entitled to an additional delegate. We doubt the advisability of accepting delegates as representatives of unaffiliated organizations, such as The American Society of Orthodontists, National Association of Examiners, Army and Navy Dental Corps, &c. These organizations, if they have any functions of professional concern, are special and need not be closely allied in any executive way with an Association which is intended only to promote the science and art of the profession. Another matter that it seems to us will greatly hinder the larger growth of the National Association is the separated membership idea. Would it not be much wiser to make membership in the National not only dependent on membership in the local, district, or state society, but likewise consequent. That is to say when a dentist joins his local, county, or district society, which is a "component" of his state society, he is then also a member of the National Association and entitled to all its privileges; and unless he is a member in good standing of his local, district, and state society he can not be a member of the National, except as an honorary member. It is proposed that the fee for the National Association membership shall be five dollars per annum. Is it not practicable to make one fee of five dollars cover all these memberships and at the same time secure sufficient revenues for all legitimate demands of the several societies? According to the best statistics now available there are at present about 14,000 members of the various state societies. If all these members paid five dollars each, and two dollars of this went to the National, and two dollars to the state, and one dollar to the component society, would not this provide sufficient funds for all necessary expenses? It is possible that the local or district society might occasionally suffer from such a division, but in these cases an emergency assessment on the members would meet any such embarassment. Under a plan of this kind the proceedings of every state society could be printed in the Association Journal, and every member would have the proceedings of every other state society as well as his own; it would be a mighty mean dentist who would not willingly join his local society to get such benefits. We believe such a proposition would meet with general favor and in five years time the National Association would have an income of at least $50,000, and be worthy of its name because of its wide constituency and the value of the work it would be capable of doing. We therefore are inclined to favor a new constitution and reorganization along the line suggested.

